# Presence and diagnostic value of circulating tsncRNA for ovarian tumor

**DOI:** 10.1186/s12943-018-0910-1

**Published:** 2018-11-22

**Authors:** Eric Y. Peng, Yang Shu, Yuke Wu, Feier Zeng, Shuangyan Tan, Yun Deng, Yiqi Deng, Haining Chen, Lei Zhu, Heng Xu

**Affiliations:** 1 State Key Laboratory of Biotherapy, West China Hospital, Sichuan University, Chengdu, Sichuan China; 2 Canyon Crest Academy, San Diego, CA USA; 3 Department of Laboratory Medicine, Precision Medicine Center, and Precision Medicine Key Laboratory of Sichuan Province, West China Hospital, Sichuan University, Chengdu, Sichuan China; 4 Department of Obstetrics and Gynecology, West China Second Hospital, Sichuan University, Chengdu, Sichuan China; 5 Department of Gastrointestinal Surgery, West China Hospital, Sichuan University, Chengdu, Sichuan China

**Keywords:** Non-coding RNA, Circulating tsncRNA, tRNA

## Abstract

tRNA-derived small non-coding RNAs (tsncRNAs), a class of newly defined small non-coding RNA, have been considered to be involved in various cellular biological processes through regulating gene expression at both transcriptional and post-transcriptional level. However, the presence of circulating tsncRNAs and their diagnostic potential is largely unclear. In this study, we investigate the serum-derived public transcriptome data from ovarian tumor patients and non-cancer controls, and find that circulating tsncRNAs cover a high proportion of total small RNA and are non-random degradation products in serum (ranging from 2.5–29.4%), which are enriched in several specific types of related tRNA (e.g., Gly-tRNA). Particularly, four tsncRNAs are differentially expressed in serum from cancer patients compared to those from healthy controls, and can predict abnormal cell proliferation with high accuracy. Our results reveal the ubiquitous presence of circulating tsncRNAs in serum, and diagnostic potential of specific tsncRNAs for ovarian tumor.

**Electronic supplementary material:**

The online version of this article (10.1186/s12943-018-0910-1) contains supplementary material, which is available to authorized users.

## Main text

Non-coding RNAs (ncRNAs), such as microRNA (miRNA) and long non-coding RNA (lncRNA), are key regulatory molecules involved in many important biological processes, including growth, development, proliferation and differentiation [1–3]. It has been demonstrated that dysregulation of cellular ncRNA leads to the initiation and progression of many types of diseases, especially for cancers [4, 5]. Emerging evidences showed that ncRNAs exist in various types of body fluids (e.g., serum) [6, 7], and specific expression patterns of some extracellular circulating ncRNA (e.g., miRNA) in different cancer types have been proposed as promising non-invasive diagnostic and prognostic biomarkers for cancers [6, 7].

tRNA-derived small non-coding RNA (tsncRNA) is a class of newly identified and defined ncRNA, including tRNA-derived fragments (tRFs), tRNA halves (tRHs) and tRNA-derived small RNAs (tsRNAs). tRFs, usually ~ 20 nt in length, are generated from mature tRNA which can be further classified into 5’ tRFs, 3’ tRFs and i’ tRF [8]. tRHs are also generated from mature tRNA which can be classified into 5’ tRHs and 3’ tRHs, with the length of ~ 30 nt. The tsRNAs are also ~ 20 nt, and generated from 3′ of pre-RNA during tRNA maturation [8, 9]. Previous studies mainly focus on the cellular tsncRNAs, and demonstrate that tsncRNAs may play a role in many human diseases [9]. However, whether tsncRNAs can be detected in the body fluid as other small ncRNAs and their diagnostic/prognostic value for diseases has not been systematically investigated. In this study, we focus on the presence of circulating tsncRNAs with the public resource (i.e., RNA-sequencing for serum RNA), including transcriptome sequencing data of serum small RNA from patients with epithelial ovarian cancer, benign tumors, borderline tumors, as well as healthy controls [10], particularly estimating the proportion of tsncRNAs in circulating ncRNAs in different individuals, and evaluating the potential of specific circulating tsncRNA as novel diagnostic risk factor for ovarian cancer.

**Fig. 1:**
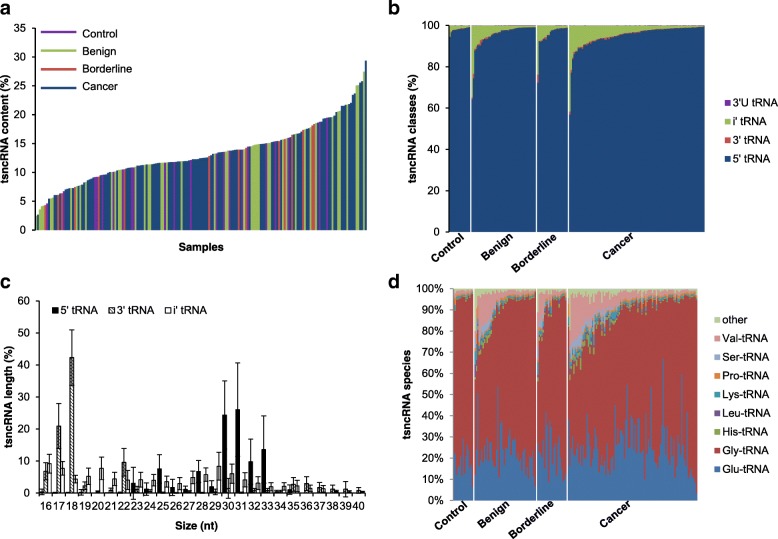
Presence and distribution of circulating tsncRNA. **a** Percentage of tsncRNA among all small RNA in serum; **b** Percentage of each type of tsncRNAs in serum; **c** Length distribution of tsncRNA from 3′, 5′ and i’ of tRNA; **d** classification of tsncRNA from different mature tRNAs

To estimate the present status and content of circulating tsncRNA, we downloaded and re-analyzed the public RNA-sequencing data [10], which cover 16–40 nt small RNA from 180 serum samples, including 15 health controls, 46 benign tumors, 22 borderline tumors, and 97 ovarian cancer patients, with totally 19 different histologic types (Additional file 1: Table S1). The sequencing reads were aligned to the mature tRNA as well as reference human genome at 100 bp downstream of tRNA genes, and can be considered as tsncRNAs according to previous reports [8]. Totally 13,544,312 reads mapped to 29,863 different types of tsncRNAs were found in all 180 samples, covering various proportions of all small RNAs (ranging from 2.5 to 29.4% with median of 12.5%) (Fig. 1a), indicating that the circulating tsncRNAs exhibit a ubiquitous presence. Next, the proportion of circulating tsncRNA was compared in different groups of individuals separately, and displayed no significant difference among the four major diagnostic types (Additional file 2: Figure S1a). With further classification in tumor, weak significance was only observed between histology of endometriod and endo/clear cell (*P* < 0.01), still with no difference of control vs. any other histologic subtypes of tumors in terms of circulating tsncRNA content (Additional file 2: Figure S1b).

Previous studies demonstrated that cellular tsncRNAs are generated by specific RNases (e.g., Dicer) rather than random degradation. Therefore, we next sought to find whether the circulating tsncRNAs have the similar characteristics (Additional file 3). After separating the circulating tsncRNAs into four groups in terms of their derivation (i.e., 3’U tRNA, i’tRNA, 3’tRNA, and 5’tRNA), 5’tRNA is the major component in all samples, followed by i’tRNA, while tsncRNAs derived from 3′ of mature tRNA (3’ tRF and 3’ tRH) and 3’ U tRNA are rare (Fig. 1b). Interestingly, the component of i’tRNA is significantly higher in circulating tsncRNAs of patients with ovarian tumors/cancer than healthy controls (*P* < 0.05), but no significant was observed among benign, borderline and cancers, indicating that enrichment of i’tRNA may reflect dysregulated cell proliferation and tumorigenesis. Next, the tsncRNAs length were also estimated in 5’tRNA, 3’tRNA and i’tRNA, and distributions of 3’ tRNA and 5’tRNA exhibited significant bias. Specifically, the large majority of 3’tRNAs are 16–18 nt in length, while 5’tRNAs are mostly 30–33 nt in length, and no significant bias length was observed for i’tRNA (Fig. 1c). However, none of these different types of tsncRNA exhibit different distribution among controls and tumors (Additional file 2: Figure S2). Additionally, we further characterized the derivation of tRNA genes, and found that most of the 5’tRNA were derived from mature Glu-, Gly-, His-, Leu-, Lys-, Pro-, Ser-, Val-tRNA (Fig. 1d). Interestingly, significantly higher component of Val-tRNA were observed in samples from ovarian cancer patients than that in healthy controls (*P* < 0.001), but not benign tumors (*P* = 0.36) or borderline tumors (*P* = 0.27). Collectively, these results indicated that the circulating tsncRNAs are ubiquitous present in serum, specific component of which are enriched in serum from patients with ovarian tumors.

**Fig. 2:**
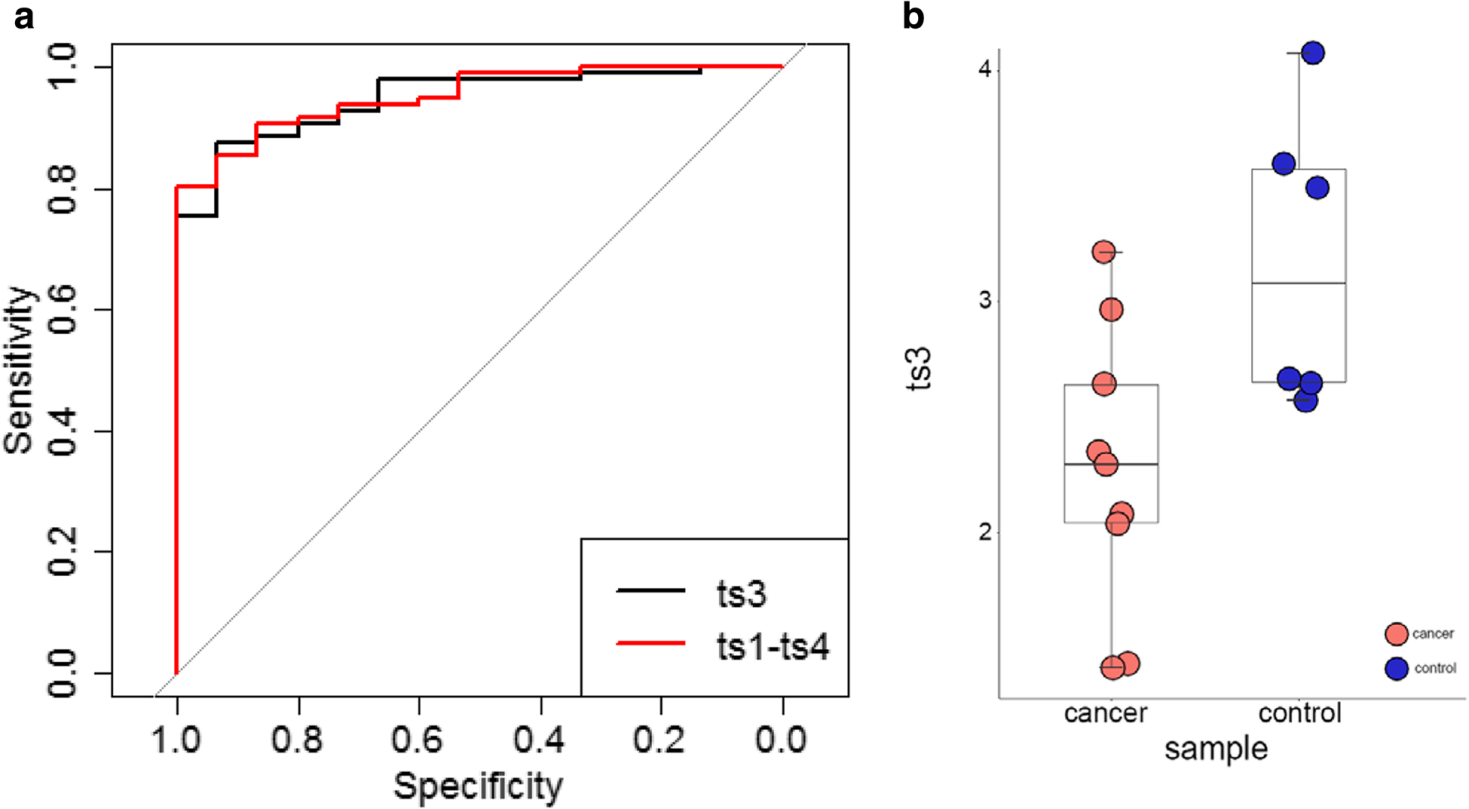
ROC analysis of tsncRNAs for ovarian cancer. **a** ROC curve analysis of ts3 and combined tsncRNAs for ovarian cancer diagnosis; **b** validation for diagnostic value of ts3 in ovary cancer and healthy controls

Next, differentially expressed circulating tsncRNAs were characterized in ovarian tumors, compared with those in health control. Only four out of 29,863 have q value < 0.01 and fold change > 2, including 2 up-regulated and 2 down-regulated, named as ts1 to ts4 (Table 1). Interestingly, all of them are derived from Gly-tRNA (Table 1), and the significance was completely lost in multivariate analyses after adjusting for ts3, indicating this tsncRNA can represent the other three. Since several types of circulating ncRNA (miRNA, lncRNA, and cirRNA) have been considered to be potential diagnostic biomarkers for multiple diseases, including cancer, we thus sought to investigate the diagnostic value of these differentially expressed circulating tsncRNAs for ovarian tumor. The Receiver Operating Characteristic (ROC) curve analysis showed that the area under the curves (AUCs) for single tscnRNA are ranging from 0.836 to 0.948 (Fig. 2a and Table 1). Interestingly, AUC values for these four tscnRNAs are still high after combining all ovarian tumors (0.828–0.946). Not surprisingly, no significant difference was observed between ts3 alone and combination of four tsncRNAs in terms of AUC value (Fig. 2a and Additional file 3), indicating only one single tsncRNA biomarker could predict ovary tumor, which may more practical than the set of 14 miRNAs described previously [10]. Moreover, validation for ts3 were conducted in ovary cancer patients and healthy female controls, exhibiting consistent significance (*P* = 0.01) (Fig. 2b). However, none of circulating tsncRNAs exhibits significant difference between ovarian cancer and benign/borderline tumors, indicating these circulating tscnRNAs exhibit diagnostic potential for ovarian tumor, which can predict abnormal cell proliferation but can’t distinguish between malignance and benign. However, Due to the limited sample size, validations are still largely needed in independent patient cohorts to establish the relationship between ts3 and ovarian cancer.

**Table 1:** Evaluation of diagnostic potential for the differentially expressed tsncRNAs

ID	Sequence information	Cancer vs. Control				Tumor vs. Control			
		FDR value^a^	Fold change	AUC	95% CI	FDR value^a^	Fold change	AUC	95% CI
ts1	GCATGGGTGGTTCAGTGGTAGAATTCTCGCCT	2.6 × 10^−3^	4.30	0.865	0.784–0.945	0.05	3.86	0.841	0.756–0.926
ts2	GCATTGGTGGTTCAATGGTAGAATTCTC	1.8 × 10^−4^	0.44	0.915	0.852–0.978	3.4 × 10^−3^	0.47	0.904	0.841–0.967
ts3	GCATTGGTGGTTCAGTGGTAGAATTCTC	3.4 × 10^−5^	0.39	0.948	0.904–0.992	3.3 × 10^−4^	0.42	0.946	0.904–0.989
ts4	TGGTTCAGTGGTAGAATTCTCGCCT	4.5 × 10^−3^	6.76	0.836	0.758–0.915	0.08	6.53	0.828	0.754–0.902
ts1-ts4	–	–	–	0.951	0.912–0.991	–	–	0.950	0.911–0.989

^a^Wilcox test was used for calculating the values of significance

Abbreviates in table: *FDR* False discovery rate, *AUC* Area under Curve, *CI* Confidence interval

## Conclusion

To our knowledge, this is the first investigation on the presence of circulating tsncRNAs and evaluation of their diagnostic value. Our studies reveal that tsncRNA is one of the major components and non-random degradation products of circulating small RNAs. Differentially expressed circulating tsncRNAs can be used as diagnostic biomarkers to predict ovarian tumorigenesis, and even other tumor types.

## Additional files

Additional file 1: **Table S1.**List of serum samples belong to each diagnostic and histology types. **Table S2.** Primers for Reverse Transcript Quantitative PCR. (DOCX 18 kb)

Additional file 2: **Figure S1.** Comparing circulating tsncRNAs content among different diagnostic and histology types. a Comparing serum tsncRNAs content among different diagnostic types. **b** circulating tsncRNAs content among different histology types. **Figure S2.** Length distribution of tsncRNA from 3′, 5′ and inter of tRNA among ovarian tumors and controls. (PDF 238 kb)

Additional file 3: Material and methods for analyses and experiments. (DOCX 16 kb)

## Abbreviations

AUC: Area under the curve
miRNA: microRNA
ncRNA: Non-coding RNA
ROC: Receiver operating characteristic
tsncRNA: tRNA-derived small non-coding RNA
