# Supplementary material for: Presence and diagnostic value of circulating tsncRNA for ovarian tumor
Source: Mol Cancer. 2018 Nov 22;17:163. doi: 10.1186/s12943-018-0910-1 (PMC6251159; doi:10.1186/s12943-018-0910-1)
Supplement: Supplementary file 1 — Table S1.List of serum samples belong to each diagnostic and histology types. Table S2. Primers for Reverse Transcript Quantitative PCR. (DOCX 18 kb) [file 12943_2018_910_MOESM1_ESM.docx]

**Additional file**

**Table S1.** List of serum samples belong to each diagnostic and histology types.

| Diagnosis | Histology | Number | Total number |
| --- | --- | --- | --- |
| Normal | Normal | 15 | 15 |
| Benign | Atypical leiomyoma | 1 | 46 |
|  | Brenner tumor | 1 |  |
|  | Brenner tumor with mucinous cystadenofibroma | 1 |  |
|  | Endometrioma | 15 |  |
|  | Fibroma | 1 |  |
|  | Fibrothecoma | 1 |  |
|  | Leiomyoma | 1 |  |
|  | Mixed Mullerian adenofibroma | 1 |  |
|  | Mucinous | 1 |  |
|  | Serous | 22 |  |
|  | Simple cyst | 1 |  |
| Borderline | Mucinous | 2 | 22 |
|  | Serous | 20 |  |
| Cancer | Clear cell | 1 | 97 |
|  | Endo/Clear Cell | 30 |  |
|  | Endometrial Adenocarcinoma | 2 |  |
|  | Endometrioid | 5 |  |
|  | Epithelioid Leiomyosarcoma | 1 |  |
|  | Granulosa Cell | 1 |  |
|  | Mucinous | 1 |  |
|  | Myxoid Leiomyosarcoma | 1 |  |
|  | Serous | 55 |  |

**Table S2. Primers for Reverse Transcript Quantitative PCR**

| **Primer name** | **primer sequence** |
| --- | --- |
| Exogenous sRNA RT-stem-loop | GTCGTATCCAGTGCAGGGTCCGAGGTATTCGCACTGGATACGACAATAGG |
| Exogenous sRNA qPCR Forward | CACGCAAGCCGCTAGCAATAC |
| ts3 RT-stem-loop | GTCGTATCCAGTGCAGGGTCCGAGGTATTCGCACTGGATACGACGAGAAT |
| ts3 qPCR Forward | CACGCAGCATTGGTGGTTCAG |
| Universal qPCR Reverse | CCAGTGCAGGGTCCGAGGTA |
