# Supplementary material for: Presence and diagnostic value of circulating tsncRNA for ovarian tumor
Source: Mol Cancer. 2018 Nov 22;17:163. doi: 10.1186/s12943-018-0910-1 (PMC6251159; doi:10.1186/s12943-018-0910-1)
Supplement: Supplementary file 2 — Figure S1. Comparing circulating tsncRNAs content among different diagnostic and histology types. a Comparing serum tsncRNAs content among different diagnostic types. b circulating tsncRNAs content among different histology types. Figure S2. Length distribution of tsncRNA from 3′, 5′ and inter of tRNA among ovarian tumors and controls. (PDF 238 kb) [file 12943_2018_910_MOESM2_ESM.pdf]

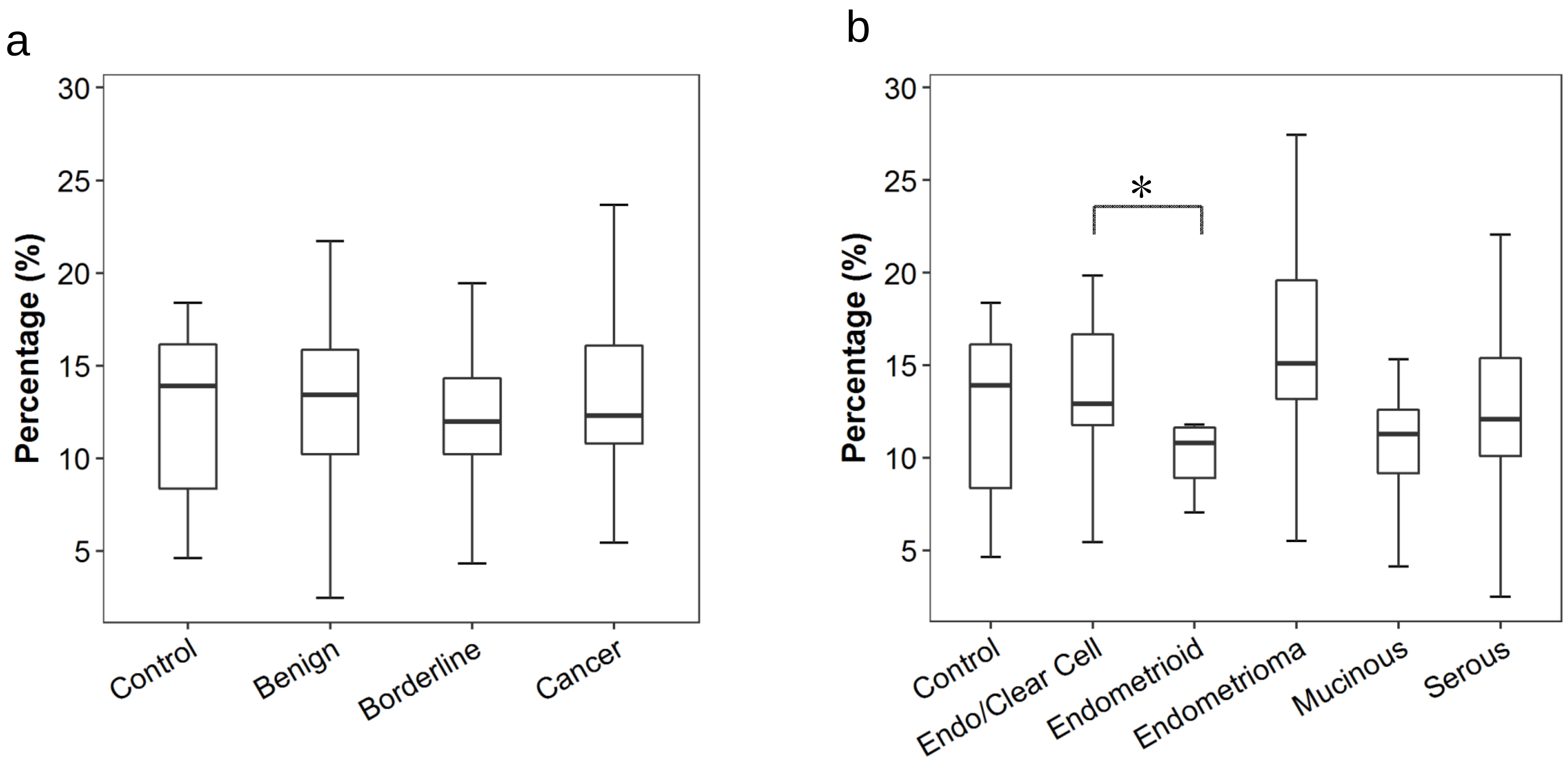

Fig S1. Comparing circulating tsncRNAs content among different diagnostic and histology types. **a** Comparing serum tsncRNAs content among different diagnostic types. **b** circulating tsncRNAs content among different histology types.

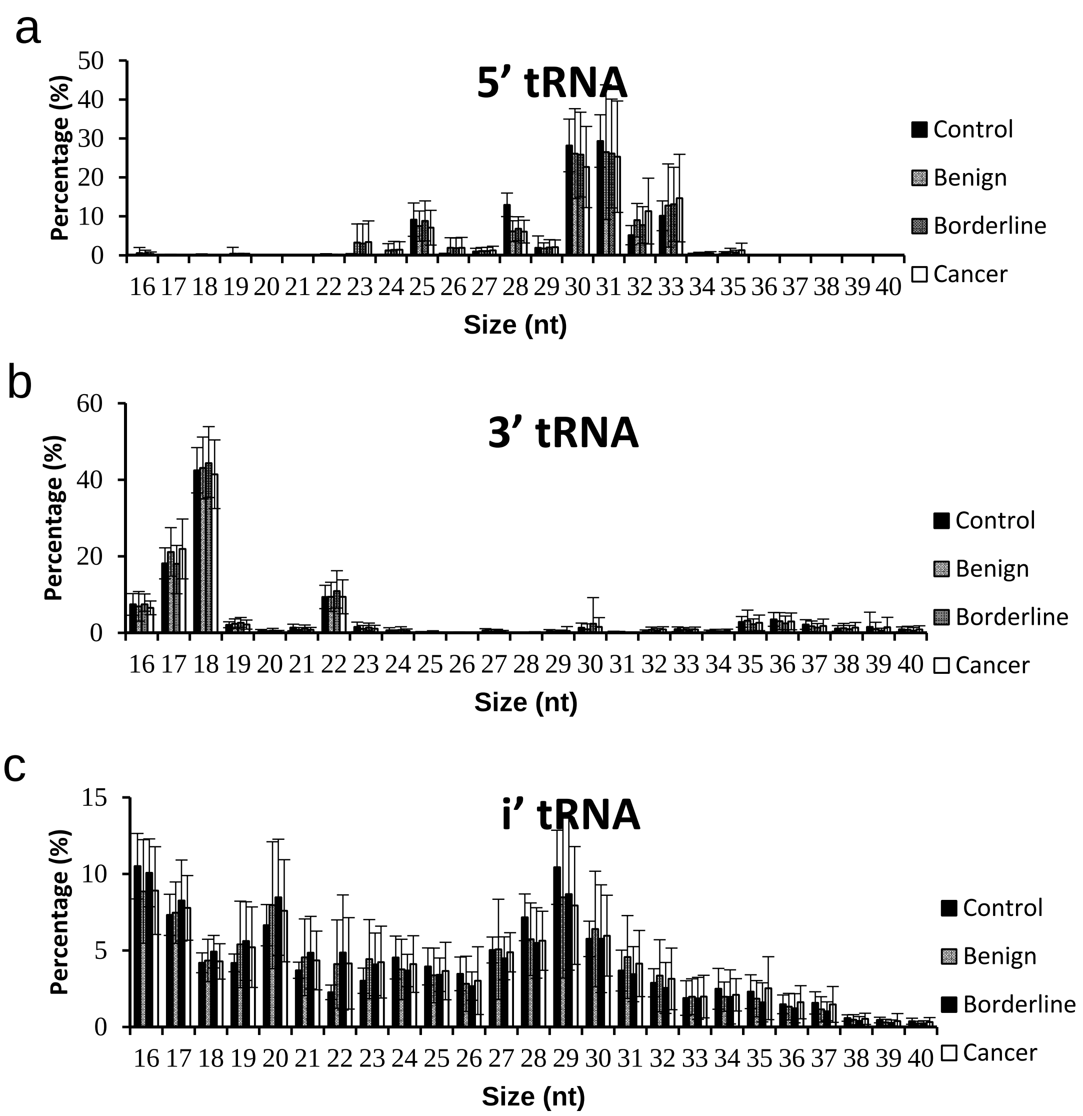

Figure S2. Length distribution of tsncRNA from 3’, 5’ and inter of tRNA among ovarian tumors and controls.
