# Supplementary material for: Presence and diagnostic value of circulating tsncRNA for ovarian tumor
Source: Mol Cancer. 2018 Nov 22;17:163. doi: 10.1186/s12943-018-0910-1 (PMC6251159; doi:10.1186/s12943-018-0910-1)
Supplement: Supplementary file 3 — Material and methods for analyses and experiments. (DOCX 16 kb) [file 12943_2018_910_MOESM3_ESM.docx]

**Material and methods**

**Source and processing of small RNA high-throughput sequencing data**

The small RNA-seq datasets of serum small RNA were retrieved from GEO database under accession number of GSE94533. After 5’ and 3’ adaptor trimming and low quality filtering, the identical reads were merged and the number of each unique reads was counted. Only 16-40 nt reads were kept for further analysis.

**Classification of small RNAs and identification of tsRNAs**

Mature tRNA genes and 100 bp downstream of tRNA genes were retrieved from UCSC (hg38). To identify tsncRNAs, 16–40 nt reads were first aligned to the mature tRNAs and downstream sequence with blast [1]. And the number of perfect matched reads was counted. We next grouped tRNA mapped reads into 5’, 3’, i’ and 3’ U of tRNA according to the positions where tsncRNAs are generated from.

**RNA extraction, reverse transcription, and quantitative realtime PCR (qRT- PCR)**

Serum from 6 health female controls and 9 ovarian cancer patients were collected from West China Hospital and West China Second Hospital, respectively. qRT-PCR of tsncRNA was conducted according to previous study [2], with synthesis sequence AGCCGCTAGCAATACACTGCCTATT as exogenous control. In brief, total RNA was extracted from 1 ml serum using the TRIzol LS reagent (Invitrogen, USA), the M-MLV Reagent Kit (Invitrogen, USA) was used for reverse transcription of tsncRNA. Real-time quantitative PCR was performed using the SYBR Premix Ex Taq (Takara Bio, China). Specific primers for tsncRNA and exogenous control were designed as shown in Table S2 (Gene Pharma, China). The data were analyzed using the comparative Ct method using synthesized sequence as an exogenous control.

**Statistical analysis**

All of the data were analyzed using R statistical environment. Will-cox test was used to evaluate the significance of differentially expressed tsncRNAs between control and tumor. To evaluate the diagnostic value of ts1-ts4 in ovarian tumor, receiver operating characteristic (ROC) curve was performed and the area under the curve (AUC) was calculated through R package “pROC”.

**Reference**

1. Altschul, S.F., et al., *Basic local alignment search tool.* J Mol Biol, 1990. **215**(3): p. 403-10.

2. Kroh, E.M., et al., *Analysis of circulating microRNA biomarkers in plasma and serum using quantitative reverse transcription-PCR (qRT-PCR).* Methods, 2010. **50**(4): p. 298-301.
